# Association between Genetic Variants and Peripheral Neuropathy in Patients with NSCLC Treated with First-Line Platinum-Based Therapy

**DOI:** 10.3390/genes14010170

**Published:** 2023-01-07

**Authors:** Corine de Jong, Gerarda J. M. Herder, Simone W. A. van Haarlem, Femke S. van der Meer, Anne S. R. van Lindert, Alexandra ten Heuvel, Jan Brouwer, Toine C. G. Egberts, Vera H. M. Deneer

**Affiliations:** 1 Department of Clinical Pharmacy, Division of Laboratories, Pharmacy, and Biomedical Genetics, University Medical Center Utrecht, 3508 GA Utrecht, The Netherlands; 2 Department of Clinical Pharmacy, St. Antonius Hospital, 3430 EM Nieuwegein, The Netherlands; 3 Department of Pulmonology, Meander Medical Center, 3813 TZ Amersfoort, The Netherlands; 4 Department of Pulmonology, St Antonius Hospital, 3430 EM Nieuwegein, The Netherlands; 5 Department of Pulmonology, Diakonessenhuis, 3582 KE Utrecht, The Netherlands; 6 Department of Pulmonology, University Medical Center Utrecht, 3508 GA Utrecht, The Netherlands; 7 Department of Pulmonology, Groene Hart Hospital, 2803 HH Gouda, The Netherlands; 8 Department of Pulmonology, Rivierenland Hospital, 4002 WP Tiel, The Netherlands; 9 Division of Pharmacoepidemiology and Clinical Pharmacology, Utrecht Institute for Pharmaceutical Sciences, Utrecht University, 3584 CG Utrecht, The Netherlands

**Keywords:** non-small cell lung cancer (NSCLC), platinum-based chemotherapy, chemotherapy-induced peripheral neuropathy (CIPN), neurotoxicity, chemotherapy-induced toxicity

## Abstract

Background: Chemotherapy-induced peripheral neuropathy (CIPN) is a common, disabling side effect in non-small cell lung cancer (NSCLC) patients treated with platinum-based therapy. There is increasing evidence for associations between genetic variants and susceptibility to CIPN. The aim of this study was to further explore genetic risk factors for CIPN by investigating previously reported genetic associations. Methods: A multicenter prospective follow-up study (PGxLUNG, NTR NL5373610015) in NSCLC patients (stage II-IV) treated with first-line platinum-based (cisplatin or carboplatin) chemotherapy was conducted. Clinical evaluation of neuropathy (CTCAE v4.03) was performed at baseline and before each cycle (four cycles, every three weeks) of chemotherapy and at three and six months after treatment initiation. The relationship between 34 single nucleotide polymorphisms (SNPs) in 26 genes and any grade (grade ≥ 1) and severe (grade ≥ 2) CIPN was assessed by using univariate and multivariate logistic regression modelling. Results: In total, 320 patients were included of which 26.3% (*n* = 84) and 8.1% (*n* = 26) experienced any grade and severe CIPN, respectively. The GG-genotype (rs879207, A > G) of TRPV1, a gene expressed in peripheral sensory neurons, was observed in 11.3% (*n* = 36) of the patients and associated with an increased risk of severe neuropathy (OR 5.2, 95%CI 2.1–12.8, adjusted *p*-value 0.012). A quarter (25%, *n* = 9/36) of the patients with the GG-genotype developed severe neuropathy compared to 6% (*n* = 17/282) of the patients with the AG- or AA-genotype. Multivariate logistic regression analysis showed statistically significant associations between the GG-genotype (ORadj 4.7, 95%CI 1.8–12.3) and between concomitant use of paclitaxel (ORadj 7.2, 95%CI 2.5–21.1) and severe CIPN. Conclusions: Patients with the GG-genotype (rs879207) of TRPV1 have an almost 5-fold higher risk of developing severe neuropathy when treated with platinum-based therapy. Future studies should aim to validate these findings in an independent cohort and to further investigated the individualization of platinum-based chemotherapy in clinical practice.

## 1. Introduction

Chemotherapy-induced peripheral neuropathy (CIPN), a disorder characterized by damage or dysfunction of the peripheral sensory nerves, is a frequently occurring, disabling and often long-lasting or even irreversible side effect of platinum-based chemotherapy [1,2]. Neuropathy manifests with clinical symptoms such as numbness, prickling or tingling in hands and feet, burning or shooting pain, muscle weakness and loss of taste [3,4]. Patients suffering from paresthesia can experience difficulties in activities of daily living, which affects patients’ quality of life to a considerable extent [5]. Frequently, CIPN may necessitate dose reduction, treatment delay, treatment switch or even early treatment termination, which may affect the disease prognosis [6,7]. As described by McWhinney et al. [8,9], the incidence and severity of neuropathy do not appear to be directly related to the response to platinum-based chemotherapy. For that reason, CIPN should be approached as an avoidable side effect of platinum-based chemotherapy [8]. Currently, no proven preventive strategies for platinum-induced neuropathy are available and clinical management is complicated by the fact that limit treatment options (e.g., duloxetine, gabapentin) are available, with only moderate effects on symptoms relief [10,11,12].

A higher cumulative dose of platinum-based chemotherapy increases the risk for CIPN; hence, symptoms of peripheral neuropathy usually occur after the second course of chemotherapy. However, neuropathy may also manifest or worsen 3–6 months after the start of platinum-based chemotherapy [1,2,13]. Patient and treatment characteristics such as pre-existing polyneuropathy, older age, diabetes mellitus, cumulative dose of chemotherapy and excessive alcohol consumption are well-known risk factors for CIPN [6,12]. In addition, genetic variants of genes involved in the development of toxicity may be of interest as predictors of benefit and harm as well. Nowadays, there is growing evidence from preclinical and clinical studies that single nucleotide polymorphisms (SNPs) are associated with susceptibility to platinum-induced peripheral sensory neuropathy [14]. Particularly, genetic variants in organic transporter molecules, DNA repair enzyme genes or genes encoding for metabolic enzymes involved in platinum detoxification are of special interest [12,15]. For example, Cecchin et al. described the association between neurotoxicity SNPs located in ATP-binding cassette, subfamily C (*ABCC*) genes in colorectal cancer patients treated with platinum-based chemotherapy and CIPN [15]. The protein encoded by *ABCC* genes are called multidrug resistance proteins and involved in the transport of substances out of cells, like platinum efflux. Other examples of genes of interest are those coding for enzymes that play an important role in detoxification (such as glutathione S-transferases) or in nucleotide excision repair pathways (such as *ERCC1*, *ERCC2*) involved in DNA repair [16]. In addition, genes expressed in peripheral sensory neurons, involved in pain sensation such transient receptor potential cation channel Subfamily V (*TRPV*), and genes that regulates neurotransmission such as calcium/calmodulin-dependent protein kinases (*CAMK*), might be of special interest as well [16]. However, previous studies investigating the contribution of genetic variants are hampered by small sample sizes and differences in clinical evaluations of neuropathy [16]. Moreover, most studies evaluating CIPN are performed in patients with colon carcinoma treated with oxaliplatin [3,15,17,18,19]. Little is known about genetic predisposition and association with CIPN in cisplatin- and carboplatin-based treatment in patients with non-small cell lung cancer (NSCLC) [20,21,22].

This study aims to further explore genetic risk factors for CIPN by investigating previously reported genetic associations in a large independent cohort of NSCLC patients treated with platinum-based chemotherapy.

## 2. Methods

### 2.1. Study Design and Patients

This study was performed as part of the PGxLUNG study, in which 350 patients were included. The study design of the PGxLUNG study has been published previously [23]. In brief, patients of the PGxLUNG study were recruited from one academic hospital (University Medical Center Utrecht), two teaching hospitals (St. Antonius Hospital Nieuwegein/Utrecht and Meander Medical Center Amersfoort) and three general hospitals (Diakonessenhuis Utrecht, Groene Hart Ziekenhuis Gouda and Ziekenhuis Rivierenland Tiel), all in the Netherlands, between February 2016 and August 2019. The inclusion criteria for this multicenter prospective follow-up study were as follows: (1) ≥18 years of age, (2) radiologically confirmed stage II-IV NSCLC, (3) planned or initiated first-line treatment with platinum-based (cisplatin or carboplatin) chemotherapy or chemoradiotherapy (according to the contemporary ESMO Clinical Practice Guidelines), and (4) previously platinum-based chemotherapy-naïve. To avoid confounding by ancestry, patients of non-European ancestry were excluded from the present study. All data were extracted from the hospitals’ electronic information systems and managed using REDCap electronic data capture tools [24].

### 2.2. Ethical Considerations

Study procedures were approved by the accredited Medical Research Ethics Committee in Nieuwegein (MEC-U, number R15.056) and implemented in accordance with the Declaration of Helsinki (64th WMA General Assembly, Fortaleza, Brazil, October 2013). The PGxLUNG study was registered on The Netherlands National Trial Register (NTR) on 26 April 2016 (NTR number NL5373610015). All patients provided written informed consent.

### 2.3. Neuropathy Phenotype

During treatment with platinum-based therapy the contemporary ESMO Clinical Practice Guidelines for diagnosis, prevention, treatment and follow-up of CIPN were taken into account [12]. Neuropathy was assessed by lung oncologists using the NCI Common Terminology Criteria for Adverse Events (CTCAE) version 4.03 definition of “Peripheral sensory neuropathy” as the categorical variable [25]. Clinical evaluation consisted of asking about typical symptoms of CIPN (such as numbness, prickling or tingling in hands and feet or loss of balance and coordination). When (severe) neuropathy was suspected, further neurological testing by a neurologist was performed at the discretion of the treating physician. Assessment of neuropathy was conducted at baseline and before each cycle (four cycles, every three weeks) of platinum-based chemotherapy and at three and six months after treatment initiation. The highest CTCAE grade within a patient between treatment initiation and the last day of follow-up was recorded, whereby neuropathy ≥ grade 2 was defined as severe neuropathy. The follow-up period for the assessment of neuropathy was six months after initiation of platinum-based chemotherapy.

### 2.4. Candidate SNPs Selection

A systematic search was performed in PubMed on 15 March 2022. The search terms included ‘platinum-based chemotherapy’, ‘pharmacogenetics’, ‘neurotoxicity’, and synonyms for each of these terms as described in Supplementary S1. Only full papers of clinical studies published in English were considered. References of the included studies were screened to identify additional studies. In addition, the online Pharmacogenomics Knowledge Base (PharmGKB) was used to identifying relevant peer-reviewed publications [26]. Genetic variants associated with CIPN caused by cisplatin or carboplatin were included when the clinical annotation levels of evidence were ‘moderate’ (level 2) or ‘high’ (level 1). In total, 73 papers were considered (see Supplementary S1). In these studies, CIPN has been graded with different instruments, such as CTCAE for peripheral sensory neuropathy, and self-reported neuropathy has been graded using the CIPN20 questionnaire scores and the scale for chemotherapy-induced long-term neurotoxicity (SCIN) [27]. From these publications, a total of 42 SNPs were selected by using a candidate SNPs approach based on the predefined criteria (see Figure S1).

### 2.5. Genotyping and Imputation

DNA samples were obtained from EDTA-blood samples using the EZ1 DNA Blood 200 μL kit (Qiagen, Hilden, Germany). DNA isolation was performed according to validated in-house protocols of the Pharmacogenetics, Pharmaceutical and Toxicological Laboratory (FarmaToxLab) of the Department of Clinical Pharmacy (ISO15189 certified), St. Antonius Hospital, Nieuwegein, the Netherlands. SNPs were genotyped by using Kompetitive allele specific PCR (KASP) at LGC Genomics (Hoddesdon, UK) and by using the Infinium Global Screening Array (GSA)-24 Kit (Illumina, San Diego, CA) at Life and Brain (Bonn, Germany). Sample quality control (QC) was performed for the genotyping by using the GSA-24 Kit with the following criteria: sample call rate > 98%, heterozygosity ±3 SD from the sample’s heterozygosity rate mean and pi-hat < 0.2 to eliminate cryptic relatedness. Genetic ethnicity was analyzed using the multidimensional scaling (MDS) approach based on Human Genome 1 K data. Standard quality control was applied for pre- and post-genotype imputation data. The following criteria were used for SNPs QC: SNP call rate > 98%, MAF > 0.05 and Hardy–Weinberg equilibrium (HWE) *p* ≥ 0.05. Imputation using these QC-passed SNPs was conducted on the University of Michigan Imputation Server [28] using the Minimac4 1.2.1, 1000 G Phase 3 v5 reference panel, GRCh37/hg19 array build and Eagle v2.4 phasing. Those SNPs with imputation quality (Rsq) > 0.8 and MAF > 0.05 were retained for association analysis. QC was performed using pLINK version 1.9 [29,30]. Since for 8 SNPs (rs113807868, rs1799735, rs1263292, rs23885, rs366631, rs56360211, rs77637129, rs830884) pre- and/or post-imputation QC were not met, in total 34 SNPs in 26 genes were included in the current study (see Figure S1) [6,7,9,15,16,18,31,32,33,34,35,36,37,38,39,40,41,42,43,44,45,46]. Table S1 shows the details (such as rsID, gene, chromosome position and functional consequence) of the selected SNPs and their distribution in the study population. Minor allele frequencies (MAFs) for the investigated SNPs were in line with those previously reported in Caucasian populations [47].

### 2.6. Potential Confounders/Effect Modifiers

The following parameters were considered to be potentially confounding and/or effect modifying variables for CIPN: age (≤70 years vs. >70 years), gender, Eastern Cooperative Oncology Group (ECOG) performance status [12,48] (ECOG PS 0 vs. ≥1), diabetes mellitus, Charlson co-morbidity index score (2–3 vs. 4–5 vs. ≥6) [12,49], concomitant chemotherapeutic agent (gemcitabine vs. paclitaxel vs. pemetrexed vs. other), platinum-agent (cisplatin vs. carboplatin), number of administered cycles of platinum-based chemotherapy, renal function (eGFR using CKD-EPI formula [50], <60 mL/min/1.73 m^2^ vs. ≥60 mL/min/1.73 m^2^), body mass index (BMI) [51] (<18.5 kg/m^2^ vs. 18.5–<25 kg/m^2^ vs. 25–<30 kg/m^2^ vs. ≥30 kg/m^2^) and tobacco use (current smoker vs. former smoker vs. non-smoker vs. unknown) [12].

### 2.7. Data Analysis

Standard summary statistics were used to describe the sample data set by using SPSS version 26.0 (IBM SPPS Statistics). The strength of the association between genetic variants and CIPN was assessed in univariate and multivariate settings with logistic regression modelling and expressed as odds ratios (OR) with corresponding 95% confidence intervals (95%CI). Associations of the individual SNPs with the neuropathy phenotype were tested in both a dominant and recessive model. The Pearson chi-square test or Fisher’s cxact test (in case the cell count in any of the tables was <5) (for categorical independent variable) was used. The false discovery rate (FDR), set at 5%, was used for correction in multiple testing based on the Benjamini–Hochberg procedure. Covariates used in the multivariate analysis were selected based on statistical significance (*p*-value < 0.10) in univariate logistic regression analysis. In addition, based on earlier described clinical significance, the number of administered cycles of platinum-based therapy was added to the multivariate model. Adjusted OR (ORadj) were calculated and a *p*-value < 0.05 (2-sided), was considered statistically significant. For the SNP with the strongest evidence for association with CIPN, the number needed to genotype (NNG) was calculated (based on the formula described by Tonk et al.) [52] to estimate the efficiency of genotyping to prevent one patient from having an adverse effect. In addition, the number needed to treat (NNT) was calculated to express the number of patients with the risk genotype who need an intervention to prevent one patient from having an adverse event [52].

## 3. Results

### 3.1. Population Characteristics

In total, 320 patients with previously untreated NSCLC, receiving platinum-based chemotherapy between April 2011 and July 2019, of the PGxLUNG study cohort (*n* = 350) [23] were included in the current analyses (30 patients excluded: 17 patients were not of European ancestry, 11 patients did not meet pre- or post-imputation QC, 2 patients died before first clinical evaluation of neuropathy at week 3). Demographic and clinical characteristics stratified by (severe) CIPN status are shown in Table 1. Median age was 65 years and 10% had diabetes. Patients received a median of three cycles (IQR 3–4) of platinum-based chemotherapy.

### 3.2. Incidence of CIPN

At treatment initiation, none of the patients were suffering from pre-existing peripheral neuropathy. In total, 26.3% (*n* = 84) patients were affected by some degree of peripheral neuropathy as assessed by the CTCAE criteria during the six months follow-up after treatment initiation. For 18.1% (*n* = 58) of the patients, grade 1 toxicity was the highest CTCAE grade recorded during follow-up. Severe neuropathy was found in 8.1% (*n* = 26) patients, with grade 2 or grade 3 toxicity presented in 7.5% (*n* = 24) and 0.6% (*n* = 2) patients, respectively. Table S2 shows the distribution of the outcome at the different follow-up moments. The highest number of patients (*n* = 36) with any grade neuropathy was assessed after administration of two cycles of platinum-based therapy. The highest number of cases (*n* = 12) of severe neuropathy was assessed three months after treatment initiation.

### 3.3. Association Analysis: Clinical Characteristics and Neuropathy

Univariate analysis showed a statistically significant association between ECOG PS at treatment initiation and neurotoxicity (ECOG ≥ 1, OR 0.5, 95%CI 0.3–0.9), as shown in Table 1. Patients treated with carboplatin/paclitaxel were at higher risk of developing both any grade (OR 8.9, 95%CI 3.3–23.7) as well as severe (OR 7.6, 95%CI 2.7–21.2) neuropathy.

### 3.4. Association Analysis: Genetic Variants and Neuropathy

To validate previously reported associations between SNPs with some aspect of CIPN, 34 selected SNPs in 26 genes were examined with the association of CTCAE for peripheral sensory neuropathy. Univariate analysis of the individual SNPs showed unadjusted statically significant associations between six SNPs and neuropathy (see Table 2 and Table S3). After multiple testing correction, the GG-genotype (rs879207, A > G) of TRPV1, a gene expressed in peripheral sensory neurons observed in 11.3% of the patients, was found to be associated with an increased risk of severe neuropathy (OR 5.2, 95%CI 2.1–12.8, FDR adjusted *p*-value 0.012). A quarter (25%, *n* = 9/36) of the patients with the GG-genotype developed severe neuropathy compared to 6% (*n* = 17/282) of patients with the AG- or AA-genotype. Within the patients with the GG-genotype, patients treated with paclitaxel (*n* = 5) experienced severe neuropathy in 80% of cases (see Table S4).

### 3.5. Multivariate Analysis: Clinical and Genetic Characteristics and Neuropathy

Multivariate analysis of genetic and clinical characteristics was performed as shown in Table 3, taking into account the GG-genotype (rs879207, A > G) of TRPV1, the number of administered cycles of platinum-based chemotherapy, ECOG PS and concomitant chemotherapeutic agents. Statistically significant association between the GG-genotype of rs879207 (ORadj 4.7, 95%CI 1.8–12.3) and between concomitant use of paclitaxel (ORadj 7.2, 95%CI 2.5–21.1) and severe CIPN were observed.

### 3.6. Population Impact Measures

The NNG and NNT for rs879207, with the GG-genotype defined as the risk genotype, were 62.2 and 7.0 for any grade neuropathy and 47.1 and 5.3 for severe neuropathy, respectively. Within patients treated with carboplatin/paclitaxel (*n* = 23), the NNG and NNT were 13.8 and 3.0 for any grade neuropathy and 8.0 and 1.7 for severe neuropathy, respectively.

## 4. Discussion

### 4.1. Main Findings

The present study demonstrates that NSCLC patients with the GG-genotype (rs879207) of *TRPV1* are at a nearly 5-fold higher risk of developing severe neuropathy when treated with platinum-based therapy. Although significant associations were found between SNPs in ABCA1 (rs2230806), ABCC2 (rs1885301, rs3740066, rs4148396) and *CAMK2N1* (rs12023000) and CIPN in univariate analyses, none of these SNPs were associated with neuropathy in multivariate setting. TRPV1 receptors are predominantly found in the nociceptive neurons of the peripheral nervous system and are involved in the transmission and modulation of pain [53]. Previously, the association between genetic predisposition of TRPV1 and CIPN was described in a cohort of ovarian cancer patients treated with carboplatin combined with paclitaxel or docetaxel [9]. In this case-control study, patients with the AG-genotype of TRPV1 (rs879207) had a 1.6-fold higher risk to develop CIPN CTCAE grade ≥ 2 as compared to non-carriers of the G-allele, while statistical significance was not reached for the comparison between patients with the AA- versus the GG-genotype. Notably, the treatment protocols and study population differed between the studies, which may have affected the risk of developing peripheral neuropathy. Although a relatively low number of the patients received paclitaxel (*n* = 23), the results of our study pointed out that the neurotoxicity was most frequent in those receiving the combination of carboplatin and paclitaxel.

Furthermore, patients with lower ECOG PS had a higher risk to develop neuropathy. This might be explained by the fact that clinicians tend to prescribe less intensive treatment regimens to patients with an impaired condition.

### 4.2. Strengths and Limitations

As a major strength of the current study, CIPN was investigated in a large independent cohort with a complete and detailed database of prospectively collected data. As a result, the quantification of the associations between CIPN, genetic variants and clinical and treatment characteristics was possible. The present study has some limitations. First, we analyzed populations of European descent only. However, the GG-genotype (rs879207) of TRPV1 is common, not only in the European population (MAF = 0.32) but also in the global population (MAF = 0.31). [54] For that reason, it is plausible that the results of the current study can be extrapolated to other populations and are most likely highly relevant for a large number of patients.

Second, although the widely-used and internationally validated CTCAE grading tool for CIPN was used, there are some concerns regarding this approach, such as the occurrence of inter-observer bias [55]. However, no substantial differences in the incidence of CIPN between patients recruited in the six different hospitals were found. Nevertheless, in general, clinicians tend to underestimate the incidence or severity of neuropathy. This may partly be caused by the fact that early symptoms are often very subtle and can easily be unnoticed if not specifically asked for [12,27,56,57]. Consequently, due to possible underreporting or underestimating of neurotoxicity by clinicians, the actual association between the GG-genotype (rs879207) of *TRPV1* and CIPN might be even stronger than has been demonstrated in our study [58,59].

### 4.3. Potential Clinical Relevance

Since recovery of CIPN is, in general, merely partial with residual symptoms in most patients, the quality of life can be reduced considerably [5]. The only proven effective measure for CIPN consists of lowering treatment intensity; therefore, the occurrence of severe neuropathy will frequently result in clinical interventions such as a dose reduction of up to 75% or early discontinuation of treatment. However, lowering treatment intensity might compromise its efficacy. Based on the results of our cohort, out of every nine patients who are genotyped, one will carry the GG-genotype of rs879207. Since our data demonstrated that carrying two copies of the minor G allele contributes to susceptibility for neuropathy, these patients are likely to benefit from further individualization of therapy. Thus, further individualization of therapy may be beneficial for at least 10% of the patients of European ancestry treated with platinum-based therapy; screening patients for the *TRPV1* (rs879207) GG-genotype could have a relevant impact on clinical practice. In addition, with a NNG of 8, we demonstrated that interventions such as dose adjustments might be considered for 12.5% of patients treated with carboplatin/paclitaxel in order to prevent severe neuropathy. Since, for advanced NSCLC patients treated with cisplatin- or carboplatin-based therapy equivalents, overall survival and response rates are reported [60], the choice of the platinum-agent should be based on expected side effects as well as the patient’s comorbidities and preferences [60].

### 4.4. Future Research

In accordance with McWhinney-Glass et al. [9] we demonstrated an association between the *TRPV1* (rs879207) GG-genotype and CIPN. Therefore, it would be of great importance to investigate this newly discovered association in an independent cohort of patients with different malignancies treated with cisplatin- or carboplatin-based therapies. In addition, further stratification according to the concomitant chemotherapeutic agent would be informative. While functional understanding of *TRPV1* is desired, the validation of our results could pave the way for a clinical intervention study. To accurately determine whether patients with the GG-genotype of (rs879207) will benefit more from an individualized regimen, a randomized controlled trial should, preferably, be performed. In this trial, the choice of the platinum-agent should take into account the *TRPV1* (rs879207) genotype with both treatment effectiveness and (neuro)toxicity as a primary endpoint.

## 5. Conclusions

This study shows that patients with the GG-genotype (rs879207) of *TRPV1* have an almost 5-fold higher risk of developing severe neuropathy when treated with platinum-based therapy. Future studies should aim to validate these likely clinically significant findings in an independent cohort. In addition, the implementation of these results in clinical practice should be investigated in clinical intervention studies with a special focus on the further individualization of platinum-based therapy to prevent the occurrence of neuropathy.

## Figures and Tables

**Table 1:** Demographic, clinical characteristics and treatment characteristics: univariate analysis of (severe) neuropathy.

	Total	Neuropathy ^#^ (≥Grade 1)			Neuropathy ^#^ (≥Grade 2)		
Characteristics		No	Yes	Crude OR (95% CI)	No	Yes	Crude OR (95% CI)
Patients, *n* (%)	320 (100)	236 (73.7)	84 (26.3)	-	294 (91.9)	26 (8.1)	-
							
Gender, *n* (%)							
Male	179 (55.9)	136 (57.6)	43 (51.2)	Ref.	166 (56.5)	13 (50.0)	Ref.
Female	141 (44.1)	100 (42.4)	41 (48.8)	1.3 (0.8–2.1)	128 (43.5)	13 (50.0)	1.3 (0.6–2.9)
							
Age at treatment initiation							
Years, mean ± SD	65.1 ± 9.3	65.0 ± 9.5	65.3 ± 8.6	1.0 (1.0–1.0)	65.1 ± 9.3	64.9 ± 8.5	1.0 (1.0–1.0)
≤70 years, *n* (%)	213 (66.6)	153 (64.8)	60 (71.4)	Ref.	193 (65.6)	20 (76.9)	Ref.
>70 years, *n* (%)	107 (33.4)	83 (35.2)	24 (28.6)	0.7 (0.4–1.3)	101 (34.4)	6 (23.1)	0.6 (0.2–1.5)
							
ECOG PS at treatment initiation, *n* (%)							
0	127 (39.7)	85 (36.0)	42 (50.0)	Ref.	114 (38.8)	13 (50.0)	Ref.
≥1	143 (44.7)	114 (48.3)	29 (34.5)	0.5 (0.3–0.9) *	132 (44.9)	11 (42.3)	0.7 (0.3–1.7)
Unknown	50(15.6)	37 (15.7)	13 (15.5)	0.7 (0.3–1.5)	48 (16.3)	2 (7.7)	0.4 (0.1–1.7)
							
Diabetes mellitus, *n* (%)							
No	288 (90.0)	213 (90.3)	75 (89.3)	Ref.	265 (90.1)	23 (88.5)	Ref.
Yes	32 (10.0)	23 (9.7)	9 (10.7)	1.1 (0.5–2.5)	29 (9.9)	3 (11.5)	1.2 (0.3–4.2)
							
Charlson co-morbidity index ^$^, *n* (%)							
2–3	108 (33.8)	77 (32.6)	31 (36.9)	Ref.	98 (33.3)	10 (38.5)	Ref.
4–5	105 (32.8)	82 (34.8)	23 (27.4)	0.7 (0.4–1.3)	98 (33.3)	7 (26.9)	0.7 (0.3–1.9)
≥6	107 (33.4)	77 (32.6)	30 (35.7)	1.0 (0.5–1.8)	98 (33.3)	9 (34.6)	0.9 (0.4–2.3)
							
Chemotherapeutic agents, first cycle, *n* (%)							
Pemetrexed	198 (61.8)	150 (63.6)	48 (57.2)	Ref.	185 (62.9)	13 (50.0)	Ref.
Gemcitabine	84 (26.3)	68 (28.8)	16 (19.0)	0.7 (0.4–1.4)	79 (26.9)	5 (19.2)	0.9 (0.3–2.6)
Paclitaxel	23 (7.2)	6 (2.5)	17 (20.2)	8.9 (3.3–23.7) *	15 (5.1)	8 (30.8)	7.6 (2.7–21.2) *
Other/unknown	15 (4.7)	12 (5.1)	3 (3.6)	0.8 (0.2–2.9)	15 (5.1)	0 (0)	0.9 (0.2–3.5)
							
Platinum-based chemotherapy, *n* (%)							
Carboplatin-based	171 (53.4)	131 (55.5)	40 (47.6)	Ref.	159 (54.1)	12 (46.2)	Ref.
Cisplatin-based	104 (32.5)	74 (31.4)	30 (35.7)	1.3 (0.8–2.3)	94 (32.0)	10 (38.5)	1.4 (0.6–3.4)
Switch cis > carbo during treatment	45 (14.1)	31 (13.1)	14 (16.7)	1.5 (0.7–3.1)	41 (13.9)	4 (15.4)	1.3 (0.4–4.2)
							
Cycles of platinum-based therapy, *n* (%)							
1	11 (3.4)	8 (3.4)	3 (3.6)	Ref.	10 (3.4)	1 (3.8)	Ref.
2	35 (10.9)	31 (13.1)	4 (4.8)	0.3 (0.1–1.9)	34 (11.5)	1 (3.8)	0.3 (0.0–5.1)
3	116 (36.6)	95 (40.3)	21 (25.0)	0.6 (0.1–2.4)	109 (37.1)	7 (26.9)	0.6 (0.1–5.8)
4	158 (49.4)	102 (43.2)	56 (66.7)	1.5 (0.4–5.7)	141 (48.0)	17 (65.5)	1.2 (0.2–10.0)
							
Renal function, baseline eGFR (CKD-EPI)							
eGFR (mL/min/1.73 m^2^), mean ± SD	83 ± 13	83 ± 14	83 ± 12	1.0 (1.0–1.0)	83 ± 13	80 ± 13	1.0 (1.0–1.0)
≥60 mL/min/1.73 m^2^, *n* (%)	294 (91.9)	214 (90.7)	80 (95.2)	Ref.	270 (91.8)	24 (92.3)	Ref.
<60 mL/min/1.73 m^2^, *n* (%)	26 (8.1)	22 (9.3)	4 (4.8)	0.5 (0.2–1.5)	24 (8.2)	2 (7.7)	0.9 (0.2–4.2)
							
BMI (kg/m^2^), *n* (%)							
18.5–<25 (normal weight)	131 (40.9)	101 (42.8)	30 (35.7)	Ref.	122 (41.5)	9 (34.6)	Ref.
<18.5 (underweight)	12 (3.8)	9 (3.8)	3 (3.6)	1.1 (0.3–4.4)	11 (3.7)	1 (3.8)	1.2 (0.1–10.7)
25–<30 (overweight)	126 (39.4)	87 (36.9)	39 (46.4)	1.5 (0.9–2.6)	113 (38.4)	13 (50.0)	1.6 (0.6–3.8)
≥30 (obese)	51 (15.9)	39 (16.5)	12 (14.3)	1.0 (0.5–2.2)	48 (16.3)	3 (11.5)	0.9 (0.2–3.3)
							
Smoking status							
Current smoker	76 (23.8)	56 (23.7)	20 (23.7)	Ref.	71 (24.1)	5 (19.2)	Ref.
Former smoker	215 (67.1)	159 (67.4)	56 (66.7)	1.0 (0.3–3.6)	196 (66.7)	19 (73.1)	1.0 (0.1–9.4)
Non-smoker	15 (4.7)	11 (4.7)	4 (4.8)	1.0 (0.5–1.8)	14 (4.8)	1 (3.8)	1.4 (0.5–3.8)
Unknown	14 (4.4)	10 (4.2)	4 (4.8)	1.1 (0.3–4.0)	13 (4.4)	1 (3.8)	1.1 (0.1–10.1)

^#^ CTCAE version 4.03 grade for peripheral sensory neuropathy between chemotherapy initiation and the last day of follow-up (six months). Clinical evaluation of neuropathy was conducted at baseline and before each cycle of platinum-based chemotherapy and, at three and six months after treatment initiation. ^$^ The Charlson comorbidity index score provides a simple means to quantify the effect of comorbid illnesses, including cardiovascular diseases, chronic obstructive pulmonary disease, liver disease and diabetes mellitus among others and accounts for the aggregate effect of multiple concurrent diseases. A higher score indicates more comorbidities. Abbreviations: BMI: body mass index; CI: confidence interval; CTCAE: Common Terminology Criteria for Adverse Events; ECOG PS: Eastern Cooperative Oncology Group Performance Status; eGFR: estimated glomerular filtration rate; OR: odds ratio; SD: standard deviation. * *p*-value < 0.05 based on independent *t*-test (for continuous independent variable) and Pearson chi-square test or Fisher’s exact test (in case the cell count in any of the tables was <5) (for categorical independent variable).

**Table 2:** Associations of SNPs with neuropathy.

Gene	rsID	Relevance to Platinum Agents/Neurotoxicity	Model	Neuropathy/Patient, %	Neuropathy Endpoint	Crude OR (95% CI)	Adjusted *p*-Value
*ABCA1*	rs2230806	Drug transporters (membrane efflux proteins)	Recessive	HT + WT (82/291), 28.2% HM (2/28), 7.1%	Any grade	Ref. 0.2 (0.1–0.9)	0.51
*ABCC2*	rs1885301		Recessive	HT + WT (77/265), 29.1% HM (7/55), 12.7%	Any grade	Ref. 0.4 (0.2–0.8)	0.53
	rs3740066		Dominant	HT + HM (39/186), 21.0% WT (45/134), 33.6%	Any grade	Ref. 0.5 (0.3–0.9)	0.42
	rs4148396		Recessive	HT + WT (74/261), 28.4% HM (8/55), 14.5%	Any grade	Ref. 0.4 (0.2–0.9)	0.44
*CAMK2N1*	rs12023000	Protein kinase in neurons, regulates neurotransmission	Recessive	HT + WT (24/317), 7.6% HM (2/3), 66.7%	Severe	Ref. 24.4 (2.1–279.1)	0.18
*TRPV1*	rs879207	Expressed in peripheral sensory neurons involved in pain sensation	Recessive	HT + WT (17/282), 6.0% HM (9/36), 25.0%	Severe	Ref. 5.2 (2.1–12.8)	0.012 *

Abbreviations: *ABCA1* or *C2*: ATP-binding cassette subfamily A, member 1 or subfamily C, member 2; *CAMK2N1*: calcium/calmodulin-dependent protein kinase II inhibitor 1; HM: homozygous minor allele; HT: heterozygous minor allele; OR: odds ratio; *TRPV1*: transient receptor potential cation channel subfamily V member 1; WT: wild type homozygous major allele.* False discovery rate adjusted *p*-value < 0.05.

**Table 3:** Multivariate analysis of (severe) neuropathy.

		Neuropathy Any Grade (≥Grade 1)		Severe Neuropathy (≥Grade 2)	
Characteristics	N (%)	Univariate Analysis ^a^ Crude OR (95% CI)	Multivariate Analysis ^b^ Adjusted OR ^c^ (95% CI)	Univariate Analysis ^a^ Crude OR (95% CI)	Multivariate Analysis ^b^ Adjusted OR ^c^ (95% CI)
Total	320 (100)	-	-	-	-
					
*TRPV1* (rs879207)					
HT + WT (*AG + AA*)	282 (88.1)	Ref.	Ref.	Ref.	Ref.
HM (*GG)*	36 (11.3)	2.0 (1.0–4.1)	1.9 (0.8–4.2)	5.2 (2.1–12.8) *	4.7 (1.8–12.3) *
					
ECOG PS at start chemotherapy					
0	127 (39.7)	Ref.	Ref.	Ref.	Ref.
≥1	143 (44.7)	0.5 (0.3–0.9) *	0.5 (0.3–0.9) *	0.7 (0.3–1.7)	0.8 (0.3–2.0)
Unknown	50(15.6)	0.7 (0.3–1.5)	0.7 (0.3–1.5)	0.4 (0.1–1.7)	0.5 (0.1–2.3)
					
Cycles of platinum-based therapy					
1	11 (3.4)	Ref.	Ref.	Ref.	Ref.
2	35 (10.9)	0.3 (0.1–1.9)	0.7 (0.1–4.6)	0.3 (0.0–5.1)	0.8 (0.0–19.7)
3	116 (36.6)	0.59 (0.1–2.4)	1.1 (0.2–5.6)	0.6 (0.1–5.8)	1.6 (0.1–21.0)
4	158 (49.4)	1.46 (0.4–5.7)	2.0 (0.4–10.5)	1.2 (0.2–10.0)	2.1 (0.2–25.1)
					
Chemotherapeutic agents, first cycle					
Pemetrexed	198 (61.8)	Ref.	Ref.	Ref.	Ref.
Gemcitabine	84 (26.3)	0.7 (0.4–1.4)	0.8 (0.4–1.7)	0.9 (0.3–2.6)	1.0 (0.4–3.0)
Paclitaxel	23 (7.2)	8.9 (3.3–23.7) *	7.5 (2.6–21.4) *	7.6 (2.7–21.2) *	7.2 (2.5–21-1) *
Other/unknown	15 (4.7)	0.8 (0.2–2.9)	0.9 (0.3–3.6)	0.9 (0.2–3.5)	-

^a^ Univariate logistic regression analysis. ^b^ Multivariate logistic regression analysis (Backward: wald). ^c^ Adjusted odds ratio: adjusted for concomitant chemotherapeutic agent, number of administered cycles of platinum-based therapy, ECOG PS and TRPV1 genotype in multivariate logistic regression analysis. Abbreviations: CI: confidence interval; ECOG PS: Eastern Cooperative Oncology Group Performance Status; HM: homozygous minor allele; HT: heterozygous minor allele; OR: odds ratio; *TRPV1*: transient receptor potential cation channel subfamily V member 1; WT: wild type (homozygote major allele).* *p*-value < 0.05.

## Data Availability

The data that support the findings of this study are available from the corresponding author upon reasonable request.

## Supplementary Materials

The following supporting information can be downloaded at: https://www.mdpi.com/article/10.3390/genes14010170/s1. Supplement S1: Search strategy and candidate SNPs selection; Figure S1: Flowchart of candidate SNPs selection; Table S1: Details and distribution of the candidate SNPs (*n* = 34); Table S2: Distribution of outcomes; Table S3: Univariate analysis of SNPs and (severe) peripheral sensory neuropathy; Table S4: Univariate and multivariate analysis of *TRPV1* genotype and concomitant therapy with paclitaxel and (severe) neuropathy.
