# Supplementary material for: Association between Genetic Variants and Peripheral Neuropathy in Patients with NSCLC Treated with First-Line Platinum-Based Therapy
Source: Genes (Basel). 2023 Jan 7;14(1):170. doi: 10.3390/genes14010170 (PMC9858836; doi:10.3390/genes14010170)
Supplement: Supplementary file 1 [file genes-14-00170-s001.zip › genes-2119013-supplementary.pdf]

Supplementary Materials

Supplement S1. Search strategy and candidate SNPs selection

Figure S1. Flowchart of candidate SNPs selection

Table S1. Details and distribution of the candidate SNPs (n=34)

Table S2. Distribution of outcomes

Table S3. Univariate analysis of SNPs and (severe) peripheral sensory neuropathy

Table S4. Univariate and multivariate analysis of *TRPV1* genotype and concomitant therapy with paclitaxel and (severe) neuropathy

Supplementary material

S1. Search strategy and candidate SNPs selection

S1.1. Search strategy

A systematic search was performed on 15 March 2022. The search terms in PubMed included 'platinum-based chemotherapy' (exposure), 'pharmacogenetics' (determinant), 'neurotoxicity' (outcome), and synonyms for each of these terms. Query: ("Cisplatin"[Mesh] OR "Cisplatin"[tiab] OR "Carboplatin"[Mesh] OR "Carboplatin"[tiab] OR "CDDP"[tiab] OR ("platinum"[tiab] AND "chemotherap*"[tiab])) AND ("Polymorphism, Genetic"[Mesh] OR (("gene"[tiab] OR "genes"[tiab] OR "genetic*"[tiab]) AND "polymorphism*"[tiab]) OR "pharmacogenomic*"[tiab] OR "SNP"[tiab] OR "SNPs"[tiab] OR "Precision Medicine"[Mesh] OR "Precision Medicine"[tiab] OR "personalized medicine"[tiab]) AND ("neurotoxic*"[tiab] OR "neuropath*"[tiab]). In total 56 publications were identified. The online Pharmacogenomics Knowledge Base (PharmGKB) was used to identifying relevant peer-reviewed publications.[26] Genetic variants associated with CIPN caused by cisplatin or carboplatin were included when the clinical annotation levels of evidence was at least 'moderate' (level 2B). No additional SNPs were added as a result of the PharmGKB search.

S1.2. Screening of publications

All publications were screened for eligibility, inclusion criteria were as follows: 1) publication in English language, 2) full-text available, 3) clinical data, 4) endpoint chemotherapy-induced peripheral neuropathy (CIPN), related to platinum-based chemotherapy. In addition, the references of the included studies were screened to identify additional studies. As shown in **Figure S1**, a total of 73 publications (56 from PubMed search, 17 from references screening) were considered.

S1.3. Candidate SNPs selection

In the current candidate SNPs selection only single nucleotide polymorphisms (SNPs) were included (no other genetic variants such as insertions, gene deletions or variations in copy numbers were selected). Inclusion criteria for SNPs were as follows: 1) SNP was statistical significantly associated with some aspect of CIPN related to platinum-based chemotherapy, 2) rsID of the SNP was published. A total of 42 SNPs associated with susceptibility to CIPN were selected through this candidate SNPs approach.

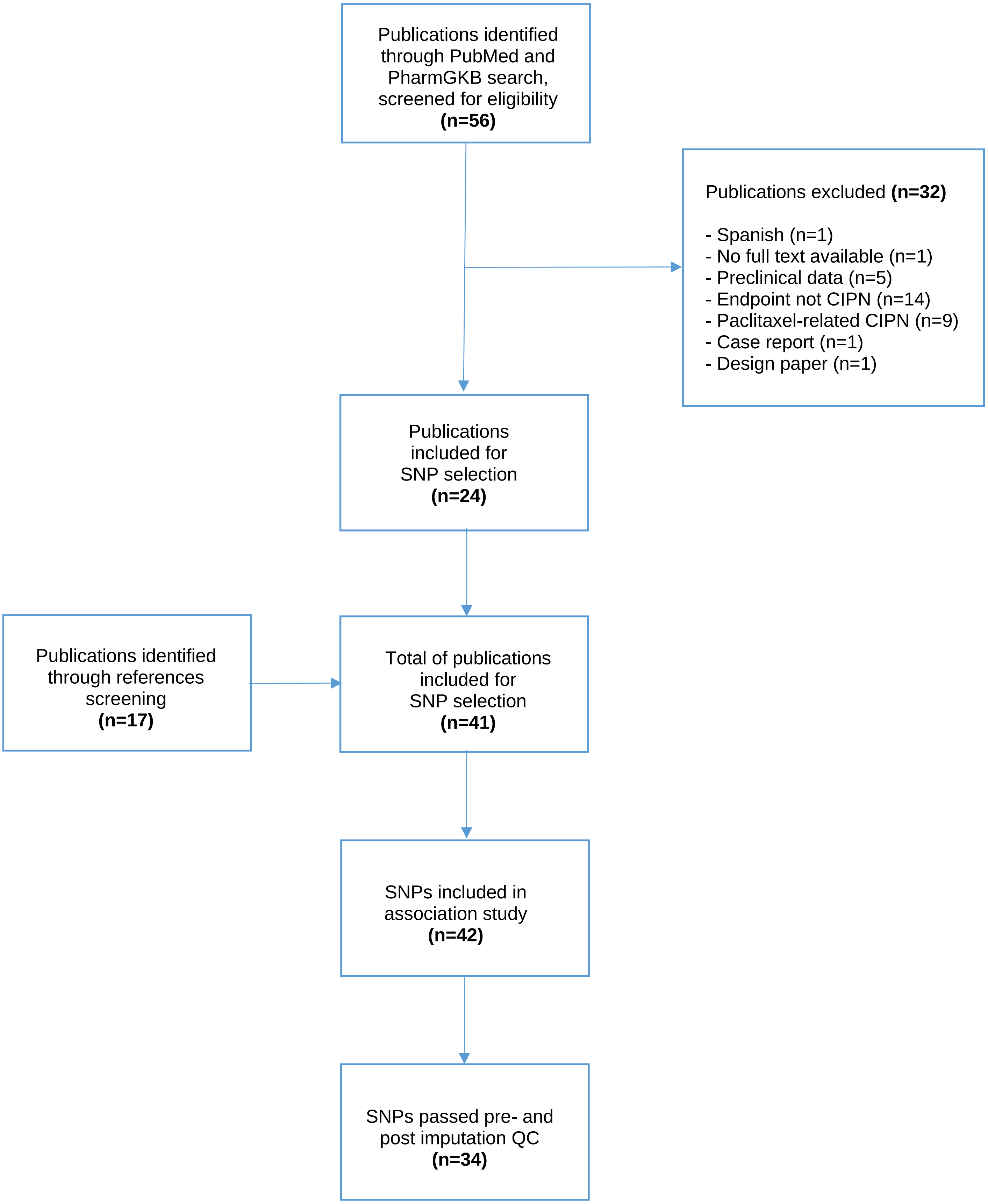

Figure S1. Flowchart of candidate SNPs selection

Table S1. Details and distribution of the candidate SNPs (n=34)

**Reference (year)**	**Relevance to platinum agents / neurotoxicity**	**Gene**	**SNP (rsID)**	**Chromosome position (GRCh37)**	**WT (%)**	**HT (%)**	**HM (%)**	**MAF**	**Missing (%)**
Hasmats (2012)	Drug transporters (membrane efflux proteins)	*ABCA1*	rs2230806	9:107620867	51.2	39.7	8.8	0.29	0.3
Ferracini (2021)		*ABCB1*	rs1045642	7:87138645	26.9	49.7	22.5	0.48	0.9
Cecchin (2013)		*ABCC1*	rs2074087	16:16184232	2.2	24.4	73.4	0.14	0
Cecchin (2013)		*ABCC2*	rs1885301	10:101541053	17.2	45.6	37.2	0.40	0
			rs3740066	10:101604207	41.9	45.3	12.8	0.35	0
			rs4148396	10:101591944	37.5	44.1	17.2	0.40	1.3
			rs717620	10:101542578	64.3	31.6	4.1	0.20	0
Johnson (2015)		*ABCC4*	rs1729786	13:95823239	11.6	18.4	1.9	0.35	68.1
Custodio (2014), Lamba (2014)		*ABCG2*	rs13120400	4:89033527	6.9	37.2	52.5	0.26	3.4
			rs3114018	4:89064581	17.2	52.5	29.4	0.44	0.9
Won (2012)	Drug metabolism and bioactivation	*ACYP2*	rs843748	2:54502912	24.7	49.7	25.0	0.50	0.6
Gamelin (2007)	Detoxification enzyme	*AGXT*	rs34116584	2:241808314	63.4	32.2	4.4	0.21	0
			rs4426527	2:241817516	62.5	33.1	4.4	0.21	0
McWhinney-Glass (2013)	Apoptosis-related protein in nerve tissues	*BCL2*	rs2849380	18:60979360	67.2	30.3	2.5	0.18	0
Won (2012)		*BTG4*	rs4936453	11:111300782	10.3	45.0	44.7	0.50	0
Avan (2015)	Protein kinase in neurons, regulates neurotransmission	*CAMK2N1*	rs12023000	1:20789645	84.7	14.4	0.9	0.08	0
Custodio (2014)	Cell cycle progression	*CCNH*	rs2230641	5:86695274	63.4	32.5	4.1	0.20	0
Inada (2010), Kim (2009)	DNA repair mechanisms	*ERCC1*	rs11615	19:45923653	41.2	47.2	11.6	0.35	0
			rs3212986	19:45912736	4.4	32.2	60.9	0.21	2.5
Lamba (2014)		*ERCC2*	rs13181	19:45854919	10.3	51.9	37.8	0.36	0
Won (2012)		*FARS2*	rs17140129	6:5298362	70.3	25.3	4.4	0.17	0
			rs6924717	6:5304851	68.0	26.3	4.1	0.18	1.6
Hong (2011), Chen (2010), Inada (2010), McLeod (2010),	Detoxification enzyme	*GSTP1*	rs1695	11:67352689	38.1	47.5	14.1	0.38	0.3

Lecomte (2006), Kumamoto (2013), Li (2010), Oldenburg (2007), Goekkurt (2009), Katayanagi (2019), Lecomte (2006), Liu (2013)									
Johnson (2015), Thomaier (2021)		*GPX7*	rs3753753	1:53069514	9.7	35.9	36.9	0.34	17.5
Antonacopoulou (2010)	Cell adhesion and cell surface-mediated signaling	*ITGB3*	rs5918	17:45360730	2.5	27.5	70.0	0.16	0
Won (2012)		*LOC105374610*	rs797519	13:51231132	2.2	55.6	2.2	0.50	40.0
Won (2012)		*LOC107986555*	rs2338	6:1573613	6.6	37.5	55.3	0.26	0.6
McWhinney-Glass (2013)		*OPRM1*	rs544093	6:154457493	1.6	17.2	81.3	0.10	0
Argyriou (2013)	Voltage-gated sodium channels	*SCN4A*	rs2302237	17:62048707	37.5	42.2	10.3	0.35	10.0
McWhinney-Glass (2013), Thomaier (2021)		*SOX10*	rs139887	22:38371396	38.1	42.2	19.7	0.41	0
Won (2012)		*TAC1*	rs10486003	7:97229778	81.0	18.1	0.9	0.10	0
McWhinney-Glass (2013)	Expressed in peripheral sensory neurons involved in pain sensation	*TRPV1*	rs879207	17:3466596	47.5	40.6	11.3	0.32	0.6
Lamba (2014)		*XPC*	rs2228001	3:14187449	36.9	47.5	15.0	0.39	0.6
Lamba (2014)		*	rs1347851	12:90566978	2.5	24.1	40.0	0.22	33.4

Abbreviations: HM, homozygous variant (homozygote minor allele); HT, heterozygous variant; NA, not available; MAF, minor allele frequency; WT, wild type (homozygote major allele). *SNPs not in a gene, selected from genome-wide association study.

ABCA1 or B1/C1/C2/C4/G2, ATP-binding cassette subfamily A, member 1 or B1/C1/C2/C4/G2; ACYP2, acylphosphatase 2, muscle type; AGXT; alanine-glyoxylate and serine-pyruvate aminotransferase; BACH2, BTB domain and CNC homolog 2; BCL2, B-cell lymphoma 2; BTG4, B-cell translocation gene 4; CAMK2N1, calcium/calmodulin-dependent protein kinase II inhibitor 1; CCNH, cyclin H; ERCC1 or 2,Excision repair cross-complementing; FARS2, phenylalanyl-tRNA synthetase 2; GSTP1, glutathione S-transferase pi 1; GPX7, glutathione peroxidase 7; ITGB3, integrin subunit beta 3; OPRM1, opioid receptor mu 1; SCN4A, sodium voltage-gated channel alpha subunit 4; SOX10, SRY-box transcription factor 10; TAC1, tachykinin precursor 1; TRPV1, transient receptor potential cation channel subfamily V member 1; XPC, xeroderma pigmentosum complementation group C.

Table S2. Distribution of outcomes

Neurotoxicity#	Follow-up 3 weeks n (%)	Follow-up 6 weeks n (%)	Follow-up 9 weeks n (%)	Follow-up 3 months n (%)	Follow-up 6 months n (%)	Highest grade during follow-up n (%)
Alive	320 (100)	317 (99.1)	314 (98.1)	306 (95.6)	281 (87.8)	-
Grade 0 (no neuropathy)#	298 (93.1)	275 (85.9)	264 (82.5)	246 (76.9)	232 (72.5)	236 (73.7)
Grade 1	21 (6.6)	27 (8.4)	23 (7.2)	19 (5.9)	14 (4,4)	58 (18.1)
Grade 2	1 (0.3)	8 (2.5)	5 (1.6)	10 (3.1)	8 (2.5)	24 (7.5)
Grade 3	0 (0)	1 (0.3)	1 (0.3)	2 (0.6)	1 (0.3)	2 (0.6)
Any grade (grade ≥1)	22 (6.9)	36 (11.3)	29 (9.1)	31 (9.7)	23 (7.2)	84 (26.3)
Severe (grade ≥2)	1 (0.3)	9 (2.8)	6 (1.9)	12 (3.8)	9 (2.8)	26 (8.1)
Missing	0 (0)	6 (1.9)	21 (6.6)	29 (9.1)	26 (8.1)	-

Neuropathy was assessed by lung oncologists using the NCI Common Terminology Criteria for Adverse Events (CTCAE) version 4.03 definition of “Peripheral sensory neuropathy”.

Table S3. Univariate analysis of SNPs and (severe) peripheral sensory neuropathy

rsID	Model	Variants	Incidence any grade (≥grade 1) neuropathy	Univariate logistic regression analysis<br><br>Neuropathy any grade (CTCAE ≥ 1) Crude OR (95% CI)	Incidence severe (≥grade 2) neuropathy	Univariate logistic regression analysis<br><br>Severe neuropathy (CTCAE ≥ 2) Crude OR (95% CI)
Total	**-**	**-**	**26.3 (84/320)**	**-**	**8.1 (26/320)**	**-**
rs1045642	Dominant	WT	25.6 (22/86)	Ref.	5.8 (5/86)	Ref.
		HM + HT	26.8 (62/231)	1.1 (0.6-1.9)	9.1 (21/231)	1.6 (0.6-4.4)
	Recessive	HT + WT	25.3 (62/245)	Ref.	7.8 (19/245)	Ref.
		HM	30.6 (22/72)	1.3 (0.7-2.3)	9.7 (7/72)	1.3 (0.5-3.2)
rs10486003	Dominant	WT	25.9 (67/259)	Ref.	7.7 (20/259)	Ref.
		HM + HT	27.9 (17/61)	1.1 (0.6-2.0)	9.8 (6/61)	1.3 (0.5-3.4)
	Recessive	HT + WT	26.2 (83/317)	Ref.	8.2 (26/317)	Ref.
		HM	33.3 (1/3)	1.4 (0.1-15.8)	0 (0/3)	-
rs11615	Dominant	WT	25.0 (33/132)	Ref.	8.3 (11/132)	Ref.
		HM + HT	27.1 (51/188)	1.1 (0.7-1.9)	8.0 (15/188)	1.0 (0.4-2.2)
	Recessive	HT + WT	26.5 (75/283)	Ref.	8.1 (23/283)	Ref.
		HM	24.3 (9/37)	0.9 (0.4-2.0)	8.1 (3/37)	1.0 (0.3-3.5)
rs12023000	Dominant	WT	26.2 (71/271)	Ref.	7.4 (20/271)	Ref.
		HM + HT	26.5 (13/49)	1.0 (0.5-2.0)	12.2 (6/49)	1.8 (0.7-4.6)
	Recessive	HT + WT	25.9 (82/317)	Ref.	7.6 (24/317)	Ref.
		HM	66.7 (2/3)	5.7 (0.5-64.1)	66.7 (2/3)	**24.4 (2.1-279.1)***
rs13120400	Dominant	WT	27.4 (46/168)	Ref.	8.3 (14/168)	Ref.
		HM + HT	25.5 (36/141)	0.9 (0.6-1.5)	8.5 (12/141)	1.0 (0.5-2.3)
	Recessive	HT + WT	27.5 (79/287)	Ref.	8.4 (24/287)	Ref.
		HM	13.6 (3/22)	0.4 (0.1-1.4)	9.1 (2/22)	1.1 (0.2-5.0)
rs13181	Dominant	WT	25.6 (31/121)	Ref.	10.7 (13/121)	Ref.
		HM + HT	26.6 (53/199)	1.1 (0.6-1.8)	6.5 (13/199)	0.6 (0.27-1.3)
	Recessive	HT + WT	25.4 (73/287)	Ref.	8.0 (23/287)	Ref.
		HM	33.3 (11/33)	1.5 (0.7-3.2)	9.1 (3/33)	1.2 (0.3-4.1)
rs1347851	Dominant	WT	22.7 (29/128)	Ref.	6.3 (8/128)	Ref.
		HM + HT	28.2 (24/85)	1.3 (0.7-2.5)	11.8 (10/85)	2.0 (0.8-5.3)
	Recessive	HT + WT	24.9 (51/205)	Ref.	8.3 (17/205)	Ref.
		HM	25.0 (2/8)	1.0 (0.2-5.1)	12.5 (1/8)	1.6 (0.2-13.6)
rs139887	Dominant	WT	25.4 (31/122)	Ref.	5.7 (7/122)	Ref.

		HM + HT	26.8 (53/198)	1.1 (0.6-1.8)	9.6 (19/198)	1.7 (0.7-4.3)
	Recessive	HT + WT	28.4 (73/257)	Ref.	8.6 (22/257)	Ref.
		HM	17.5 (11/63)	0.5 (0.3-1.1)	6.3 (4/63)	0.7 (0.2-2.2)
rs1695	Dominant	WT	25.4 (31/122)	Ref.	7.4 (9/122)	Ref.
		HM + HT	26.9 (53/197)	1.1 (0.7-1.8)	8.6 (17/197)	1.2 (0.5-2.8)
	Recessive	HT + WT	25.5 (70/274)	Ref.	7.7 (21/274)	Ref.
		HM	31.1 (14/45)	1.3 (0.7-2.6)	11.1 (5/45)	1.5 (0.5-4.2)
rs17140129	Dominant	WT	23.6 (53/225)	Ref.	7.6 (17/225)	Ref.
		HM + HT	32.6 (31/95)	1.6 (0.9-2.7)	9.5 (9/95)	1.3 (0.6-3.0)
	Recessive	HT + WT	26.1 (80/306)	Ref.	8.5 (26/306)	Ref.
		HM	28.6 (4/14)	1.1 (0.4-3.7)	0 (0/14)	-
rs1729786	Dominant	WT	21.6 (8/37)	Ref.	5.4 (2/37)	Ref.
		HM + HT	24.6 (16/65)	1.2 (0.5-3.1)	12.3 (8/65)	2.5 (0.5-12.2)
	Recessive	HT + WT	25.0 (24/96)	Ref.	10.4 (10/96)	Ref.
		HM	0 (0/6)	-	0 (0/6)	-
rs1885301	Dominant	WT	26.9 (32/119)	Ref.	8.4 (10/119)	Ref.
		HM + HT	25.9 (52/201)	1.0 (0.6-1.6)	8.0 (16/201)	0.9 (0.4-2.2)
	Recessive	HT + WT	29.1 (77/265)	Ref.	8.7 (23/265)	Ref.
		HM	12.7 (7/55)	**0.4 (0.2-0.8)***	5.5 (3/55)	0.6 (0.2-2.1)
rs2074087	Dominant	WT	25.5 (60/235)	Ref.	8.5 (20/235)	Ref.
		HM + HT	28.2 (24/85)	1.2 (0.7-2.0)	7.1 (6/85)	0.8 (0.3-2.1)
	Recessive	HT + WT	26.2 (82/313)	Ref.	8.0 (25/313)	Ref.
		HM	28.6 (2/7)	1.1 (0.2-5.9)	14.3 (1/7)	1.9 (0.2-16.6)
rs2228001	Dominant	WT	26.1 (31/119)	Ref.	10.9 (13/119)	Ref.
		HM + HT	26.4 (53/201)	1.0 (0.6-1.7)	6.5 (13/201)	0.6 (0.3-1.3)
	Recessive	HT + WT	27.9 (76/272)	Ref.	8.8 (24/272)	Ref.
		HM	16.7 (8/48)	0.5 (0.2-1.2)	4.2 (2/48)	0.5 (0.1-2.0)
rs2230641	Dominant	WT	28.6 (58/203)	Ref.	8.4 (17/203)	Ref.
		HM + HT	22.2 (26/117)	0.7 (0.4-1.2)	7.7 (9/117)	0.9 (0.4-2.1)
	Recessive	HT + WT	26.7 (82/307)	Ref.	7.8 (24/307)	Ref.
		HM	15.4 (2/13)	0.5 (0.1-2.3)	15.4 (2/13)	2.1 (0.5-10.2)
rs2230806	Dominant	WT	23.8 (39/164)	Ref.	8.5 (14/164)	Ref.
		HM + HT	29.0 (45/155)	1.3 (0.8-2.2)	7.7 (12/155)	0.9 (0.4-2.0)
	Recessive	HT + WT	28.2 (82/291)	Ref.	8.6 (25/291)	Ref.
		HM	7.1 (2/28)	**0.2 (0.1-0.9)***	3.6 (1/28)	0.4 (0.1-3.0)
rs2302237	Dominant	WT	26.7 (32/120)	Ref.	5.0 (6/120)	Ref.
		HM + HT	23.8 (40/168)	0.9 (0.5-1.5)	9.5 (16/168)	2.0 (0.8-5.3)

	Recessive	HT + WT	25.1 (64/255)	Ref.	7.1 (18/255)	Ref.
		HM	24.2 (8/33)	1.0 (0.4-2.2)	12.1 (4/33)	1.8 (0.6-5.7)
rs2338	Dominant	WT	27.7 (49/177)	Ref.	9.0 (16/177)	Ref.
		HM + HT	24.1 (34/141)	0.8 (0.5-1.4)	6.4 (9/141)	0.7 (0.3-1.6)
	Recessive	HT + WT	26.6 (79/297)	Ref.	8.9 (24/297)	Ref.
		HM	19.0 (4/21)	0.7 (0.2-2.0	4.8 (1/21)	0.6 (0.1-4.4)
rs2849380	Dominant	WT	26.5 (57/215)	Ref.	9.3 (20/215)	Ref.
		HM + HT	25.7 (27/105)	1.0 (0.6-1.6)	5.7 (6/105)	0.6 (0.2-1.5)
	Recessive	HT + WT	26.6 (83/312)	Ref.	8.0 (25/312)	Ref.
		HM	12.5 (1/8)	0.4 (0.1-3.3)	12.5 (1/8)	1.6 (0.2-13.9)
rs3114018	Dominant	WT	33.0 (31/94)	Ref.	9.6 (9/94)	Ref.
		HM + HT	23.3 (52/223)	0.6 (0.4-1.1)	7.6 (17/223)	0.8 (0.3-1.8)
	Recessive	HT + WT	25.6 (67/262)	Ref.	8.0 (21/262)	Ref.
		HM	29.1 (16/55)	1.2 (0.6-2.3)	9.1 (5/55)	1.2 (0.4-3.2)
rs3212986	Dominant	WT	26.2 (51/195)	Ref.	9.2 (18/195)	Ref.
		HM + HT	26.5 (31/117)	1.0 (0.6-1.7)	6.8 (8/117)	0.7 (0.3-1.7)
	Recessive	HT + WT	26.2 (78/298)	Ref.	8.1 (24/298)	Ref.
		HM	28.6 (4/14)	2.0 (0.9-4.1)	14.3 (2/14)	1.9 (0.4-9.0
rs34116584	Dominant	WT	25.6 (52/203)	Ref.	6.4 (13/203)	Ref.
		HM + HT	27.4 (32/117)	1.1 (0.7-1.8)	11.1 (13/117)	1.8 (0.8-4.1)
	Recessive	HT + WT	25.8 (79/306)	Ref.	6.9 (24/306)	Ref.
		HM	35.7 (5/14)	0.4 (0.2-1.1)	14.3 (2/14)	0.6 (0.1-2.4)
rs3740066	Dominant	WT	33.6 (45/134)	Ref.	11.2 (15/134)	Ref.
		HM + HT	21.0 (39/186)	**0.5 (0.3-0.8)***	5.9 (11/186)	0.5 (0.2-1.1)
	Recessive	HT + WT	28.0 (78/279)	Ref.	8.6 (24/279)	Ref.
		HM	14.6 (6/41)	0.4 (0.2-1.1)	4.9 (2/41)	0.6 (0.1-2.4)
rs3753753	Dominant	WT	31.4 (37/118)	Ref.	9.3 (11/118)	Ref.
		HM + HT	23.3 (34/146)	0.7 (0.4-1.2)	6.8 (10/146)	0.7 (0.3-1.8)
	Recessive	HT + WT	28.8 (67/233)	Ref.	9.0 (21/233)	Ref.
		HM	12.9 (4/31)	0.4 (0.1-1.1)	0 (0/31)	-
rs4148396	Dominant	WT	30.8 (37/120)	Ref.	9.2 (11/120)	Ref.
		HM + HT	23.0 (45/196)	0.7 (0.4-1.1)	7.1 (14/196)	0.8 (0.3-1.7)
	Recessive	HT + WT	28.4 (74/261)	Ref.	8.4 (22/261)	Ref.
		HM	14.5 (8/55)	**0.4 (0.2-0.9)***	5.5 (3/55)	0.6 (0.2-2.2)
rs4388268	Dominant	WT	24.4 (51/209)	Ref.	9.1 (19/209)	Ref.
		HM + HT	30.4 (31/102)	1.4 (0.8-2.3)	5.9 (6/102)	0.6 (0.2-1.6)
	Recessive	HT + WT	26.0 (79/304)	Ref.	8.2 (25/304)	Ref.

		HM	42.9 (3/7)	2.1 (0.5-9.8)	0 (0/7)	-
rs4426527	Dominant	WT	25.0 (50/200)	Ref.	6.5 (13/200)	Ref.
		HM + HT	28.3 (34/120)	1.2 (0.7-2.0)	10.8 (13/120)	1.8 (0.8-3.9)
	Recessive	HT + WT	25.8 (79/306)	Ref.	7.8 (24/306)	Ref.
		HM	35.7 (5/14)	1.6 (0.5-4.9)	14.3 (2/14)	2.0 (0.4-9.3)
rs4936453	Dominant	WT	26.6 (38/143)	Ref.	7.0 (10/143)	Ref.
		HM + HT	26.0 (46/177)	1.0 (0.6-1.6)	9.0 (16/177)	1.3 (0.6-3.0)
	Recessive	HT + WT	26.1 (75/287)	Ref.	8.4 (24/287)	Ref.
		HM	27.3 (9/33)	1.1 (0.5-2.4)	6.1 (2/33)	0.7 (0.2-3.1)
rs544093	Dominant	WT	27.7 (72/260)	Ref.	8.8 (23/260)	Ref.
		HM + HT	20.0 (12/60)	0.7 (0.3-1.3)	5.0 (3/60)	0.5 (0.2-1.9)
	Recessive	HT + WT	26.7 (84/315)	Ref.	8.3 (26/315)	Ref.
		HM	0 (0/5)	-	0 (0/5)	-
rs5918	Dominant	WT	25.4 (57/224)	Ref.	7.6 (17/224)	Ref.
		HM + HT	28.1 (27/96)	1.2 (0.7-2.0)	9.4 (9/96)	1.3 (0.5-2.9)
	Recessive	HT + WT	26.3 (82/312)	Ref.	8.3 (26/312)	Ref.
		HM	25.0 (2/8)	0.9 (0.2-4.7)	0 (0/8)	-
rs6924717	Dominant	WT	26.5 (58/219)	Ref.	7.3 (16/219)	Ref.
		HM + HT	27.1 (26/96)	1.0 (0.6-1.8)	10.4 (10/96)	1.5 (0.6-3.4)
	Recessive	HT + WT	27.1 (82/303)	Ref.	8.6 (26/303)	Ref.
		HM	16.7 (2/12)	0.5 (0.1-2.5)	0 (0/12)	-
rs717620	Dominant	WT	29.6 (61/206)	Ref.	8.7 (18/206)	Ref.
		HM + HT	20.2 (23/114)	0.6 (0.4-1.0)	7.0 (8/114)	0.8 (0.3-1.9)
	Recessive	HT + WT	27.0 (83/307)	Ref.	8.1 (25/307)	Ref.
		HM	7.7 (1/13)	0.2 (0.0-1.8)	7.7 (1/13)	0.9 (0.1-7.5)
rs797519	Dominant	WT	14.3 (1/7)	Ref.	0 (0/7)	Ref.
		HM + HT	28.6 (53/185)	2.4 (0.3-20.5)	7.6 (14/185)	-
	Recessive	HT + WT	28.1 (52/185)	Ref.	7.0 (13/185)	Ref.
		HM	28.6 (2/7)	1.0 (0.2-5.4)	14.2 (1/7)	2.2 (0.3-19.7)
rs843748	Dominant	WT	21.5 (17/79)	Ref.	10.1 (8/79)	Ref.
		HM + HT	27.6 (66/239)	1.4 (0.8-2.6)	7.5 (18/239)	0.7 (0.3-1.7)
	Recessive	HT + WT	22.5 (18/80)	Ref.	5.0 (4/80)	Ref.
		HM	27.3 (65/238)	0.8 (0.4-1.4)	9.2 (22/238)	0.5 (0.2-1.6)
rs879207	Dominant	WT	25.0 (38/152)	Ref.	7.2 (11/152)	Ref.
		HM + HT	27.1 (45/166)	1.1 (0.7-1.8)	9.0 (15/166)	1.3 (0.6-2.9)
	Recessive	HT + WT	24.5 (69/282)	Ref.	6.0 (17/282)	Ref.
		HM	38.9 (14/36)	2.0 (1.0-4.1)	25.0 (9/36)	**5.2 (2.1-12.8)***

Abbreviations: CI: confidence interval; CTCAE: Common Terminology Criteria for Adverse Events; OR: Odds ratio; HM: homozygous variant (homozygote minor allele); HT: heterozygous variant; WT: wild type (homozygote major allele).

*p-value < 0.05.

Table S4. Univariate and multivariate analysis of *TRPV1* genotype and concomitant therapy with paclitaxel and (severe) neuropathy

	**Incidence any grade (≥grade 1) neuropathy**	**Univariate analysis[a]<br><br>Crude OR (95%CI)**	**Multivariate analysis[b]<br><br>Adjusted OR (95%CI)**	**Incidence severe (≥grade 2) neuropathy**	**Univariate analysis[a]<br><br>Crude OR (95%CI)**	**Multivariate analysis[b]<br><br>Adjusted OR (95%CI)**
HT+WT (*AG+AA*), no paclitaxel	21.6% (57/264)	Ref.	Ref.	4.9% (13/264)	Ref.	Ref.
HM (*GG),* no paclitaxel	29.0% (9/31)	1.5 (0.7-3.4)	1.5 (0.7-3.5)	16.1% (5/31)	3.7 (1.2-11.2)*****	3.7 (1.2-11.5)*****
HT+WT (*AG+AA*), paclitaxel	66.7% (12/18)	7.3 (2.6-20.2)*****	6.1 (2.1-17.6)*****	22.2% (4/18)	5.5 (1.6-19.1)*****	5.0 (1.4-18.2)*****
HM (*GG),* paclitaxel	100% (5/5)	-	-	80% (4/5)	77.2 (8.1-740.9)*****	70.5 (5.9-837.7)*****

[a] Univariate logistic regression analysis.
[b] Multivariate logistic regression analysis (Backward: wald).
[c] Adjusted odds ratio: adjusted for the number of administered cycles of platinum-based therapy and ECOG PS in multivariate logistic regression analysis.

Abbreviation: CI: confidence interval; HM: homozygous minor allele; HT: heterozygous major allele; OR: odds ratio; *TRPV1*: transient receptor potential cation channel subfamily V member 1; WT: wild type, homozygous major allele.
* p-value < 0.05
